# 7-HYB, a Phenolic Compound Isolated from *Myristica fragrans* Houtt Increases Cell Migration, Osteoblast Differentiation, and Mineralization through BMP2 and β-catenin Signaling

**DOI:** 10.3390/ijms21218059

**Published:** 2020-10-29

**Authors:** Kyung-Ran Park, Yoon-Ju Kwon, Ji-Eun Park, Hyung-Mun Yun

**Affiliations:** 1 Department of Oral and Maxillofacial Pathology, School of Dentistry, Kyung Hee University, Seoul 02447, Korea; rudfks282@naver.com; 2 National Institute for Korean Medicine Development, Gyeongsan 38540, Korea; mars005@nikom.or.kr (Y.-J.K.); soalsdn728@nikom.or.kr (J.-E.P.)

**Keywords:** *Myristicafragrans* Houtt, 7-HYB, osteoblast, BMP2, β-catenin

## Abstract

The seeds (nutmegs) of *Myristica fragrans* Houtt have been used as popular spices and traditional medicine to treat a variety of diseases. A phenolic compound, ((7S)-8′-(benzo[3′,4′]dioxol-1′-yl)-7-hydroxypropyl)benzene-2,4-diol (7-HYB) was isolated from the seeds of *M. fragrans*. This study aimed to investigate the anabolic effects of 7-HYB in osteogenesis and bone mineralization. In the present study, 7-HYB promotes the early and late differentiation of MC3T3-E1 preosteoblasts. 7-HYB also elevated cell migration rate during differentiation of the preosteoblasts with the increased phosphorylation of mitogen-activated protein kinases (MAPKs) including ERK1/2, p38, and JNK. In addition, 7-HYB induced the protein level of BMP2, the phosphorylation of Smad1/5/8, and the expression of RUNX2. 7-HYB also inhibited GSK3β and subsequently increased the level of β-catenin. However, in bone marrow macrophages (BMMs), 7-HYB has no biological effects in cell viability, TRAP-positive multinuclear osteoclasts, and gene expression (c-Fos and NF-ATc1) in receptor activator of NF-κB ligand (RANKL)-induced osteoclastogenesis. Our findings suggest that 7-HYB plays an important role in osteoblast differentiation through the BMP2 and β-catenin signaling pathway. It also indicates that 7-HYB might have a therapeutic effect for the treatment of bone diseases such as osteoporosis and periodontitis.

## 1. Introduction

Bone is a dynamically mineralized connective tissue that is broken down and re-formed throughout life through complex events [1,2]. Bone metabolism is dependent on the balance between osteoblast-mediated formation and osteoclast-mediated bone resorption during the physiological process of bone remodeling in the adult skeleton [3,4]. Metabolic bone diseases such as osteoporosis and periodontitis are mainly characterized by defective or excessive bone formation, and these pathogenesis are caused by dysregulation in the commitment, differentiation, and survival of osteoblast lineages as well as the impaired differentiation and function of osteoclasts [5,6]. However, the limitation of safe and efficient drugs regulating the number and function of osteoblasts and osteoclasts makes it much more difficult to treat bone diseases [5,7,8]. In this context, it is important to identify potential compounds based on the molecular and cellular mechanisms underlying pathogenesis to translate this knowledge into efficient bone disease therapy.

*Myristica fragrans* Houtt. belongs to the Myristicaceae family. Its seed (nutmeg) has been widely used as popular spices, sweet cooking, and a variety of drinks [9]. Nutmeg has been also used as traditional medicine such as rheumatism, cholera, psychosis, stomach cramps, nausea, diarrhea, flatulence, and anxiety [10,11]. Nutmeg includes fixed and essential oil, triterpenes, and various types of phenolic compounds, exhibiting biological activities that support its use in traditional medicine [12]. Several phenolic compounds showed anti-inflammatory, anxiolytic, and antioxidative activities [13,14,15]. ((7S)-8′-(benzo [3′,4′]dioxol-1′-yl)-7-hydroxypropyl)benzene-2,4-diol (7-HYB) isolated from the seeds of *M. fragrans* is a phenolic compound. However, its biological effects in osteoblast and osteoclast differentiation have not been defined yet.

In the present study, we examined intracellular signaling and mechanisms underlying the biological function of 7-HYB on the survival, migration, and differentiation of MC3T3-E1 preosteoblasts and bone marrow macrophages (BMMs) as an in vitro cell system. Our data present 7-HYB as a potential phenolic compound to treat bone diseases such as osteoporosis and periodontitis.

## 2. Results

### 2.1. 7-HYB Has No Effect on the Cell Toxicity in Preosteoblasts

**Figure 1:**
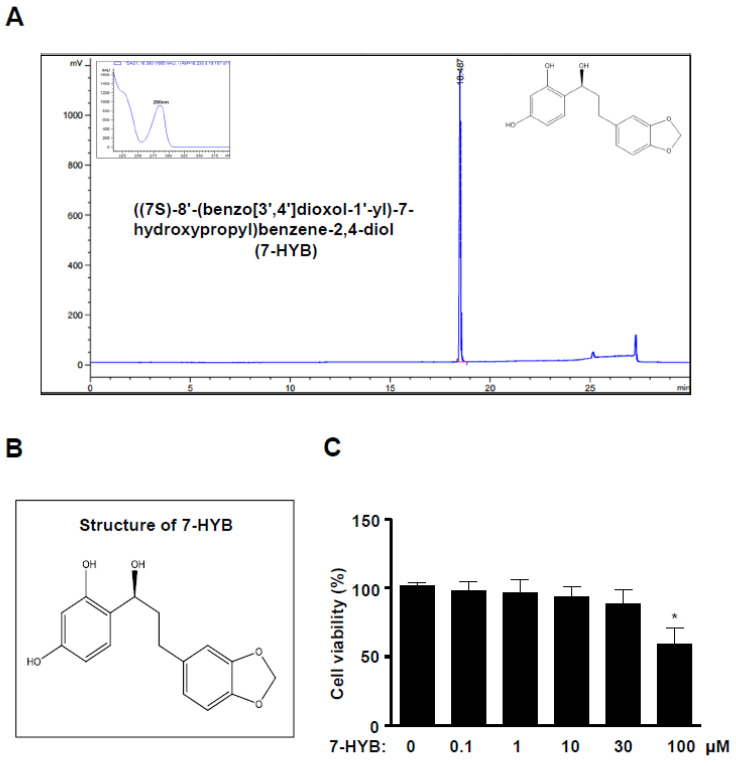
Effect of 7-HYB on the cytotoxicity of preosteoblasts. (**A**) HPLC chromatogram of ((7S)-8′-(benzo[3′,4′]dioxol-1′-yl)-7-hydroxypropyl)benzene-2,4-diol (7-HYB) isolated from the seeds of *M. fragrans*. (**B**) Chemical structure of 7-HYB. (**C**) Preosteoblasts were cultured in 0.1, 1, 10, 30, and 100 μM of 7-HYB for 24 h, and cell viability was measured by the MTT assay. Data represent the mean ± S.E.M. of experiments. *, *p* < 0.05: statistically significant difference when compared to the control.

((7S)-8′-(benzo[3′,4′]dioxol-1′-yl)-7-hydroxypropyl)benzene-2,4-diol (7-HYB) was isolated from the seeds of *Myristica fragrans* and the HPLC chromatogram and structure of 7-HYB are shown in Figure 1A,B. To test the effects of 7-HYB on the viability of preosteoblasts, the cells were treated with 0.1–100 μM 7-HYB for 24 h. 7-HYB did not affect cell viability except for 100 μM (Figure 1C). For the following experiments, we used the dose of 7-HYB below 100 μM.

### 2.2. 7-HYB Promotes the Early Osteoblast Differentiation of Preosteoblasts

**Figure 2:**
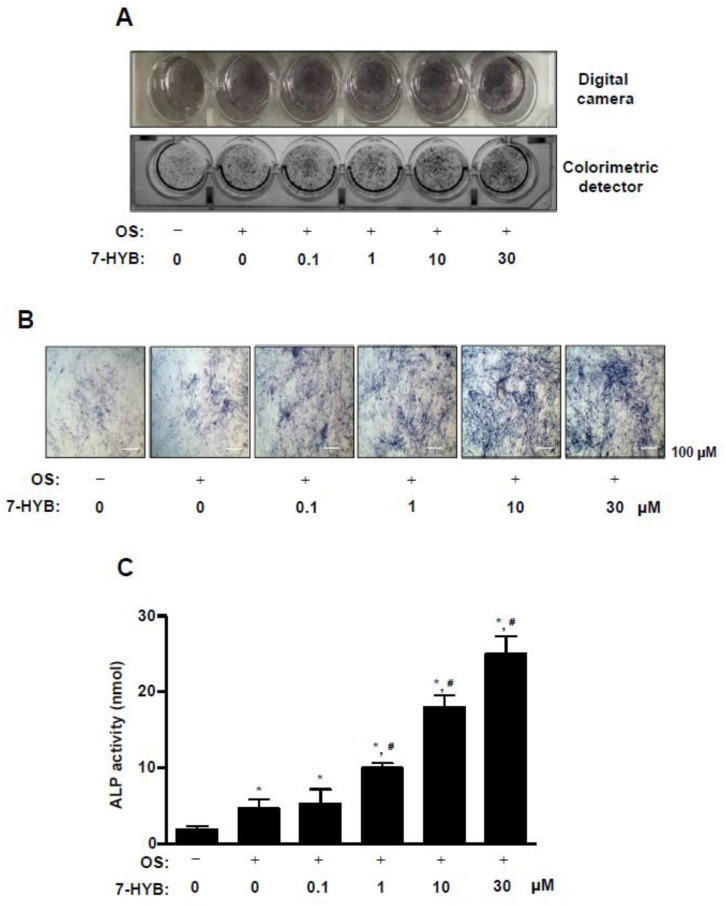
Effect of 7-HYB on the early osteoblast differentiation. (**A**,**B**) Preosteoblasts were cultured in osteogenic supplement medium (OS) containing 50 μg/mL L-ascorbic acid (L-AA) and 10 mM β-glycerophosphate (β-GP) with 0.1, 1, 10, and 30 μM of 7-HYB for seven days. The staining of alkaline phosphatase (ALP) was detected using a digital camera (*top*) and colorimetric detector (*bottom*) (A), the individual ALP-stained cells were observed under a light microscope (B). (**C**) ALP activity was measured at 405 nm by the Multiskan GO Microplate Spectrophotometer. Scale bar: 100 μm. Data represent the mean ± S.E.M. of experiments. *, *p* < 0.05: statistically significant difference when compared to the control. #, *p* < 0.05: statistically significant difference when compared to OS.

In order to investigate whether 7-HYB affects osteoblast differentiation, 7-HYB was treated with osteogenic supplement medium (OS) containing 50 μg/mL L-ascorbic acid (L-AA) and 10 mM β-glycerophosphate (β-GP) for seven days. Alkaline phosphatase (ALP) staining was observed to detect the early differentiation of preosteoblasts using a digital camera and colorimetric detector. The ALP staining showed that 7-HYB promoted the early osteoblasts differentiation in a dose dependent manner (Figure 2A). Using a light microscope, we also confirmed that 7-HYB increased ALP-stained cells in a dose dependent manner (Figure 2B). In addition, 7-HYB also significantly elevated the ALP enzymatic activity in a dose dependent manner, which was similar to results of ALP staining (Figure 2C).

### 2.3. 7-HYB Enhances the Late Osteoblast Differentiation of Preosteoblasts

**Figure 3:**
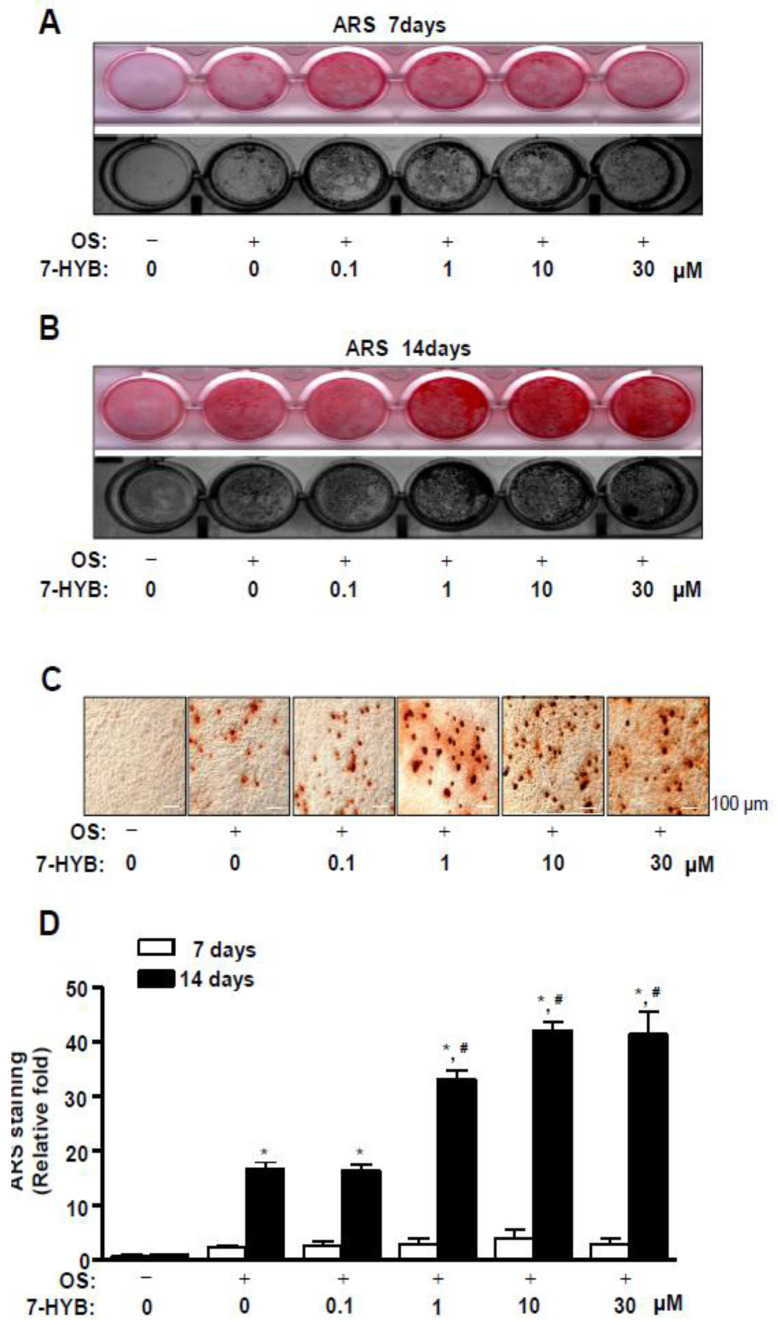
Effect of 7-HYB on the late osteoblast differentiation. (**A–D**) Preosteoblasts were cultured in OS with 0.1, 1, 10, and 30 μM of 7-HYB, and mineralized nodule formation was assessed by ARS staining at seven days (**A**) and 14 days (**B**). The staining of ARS was detected using a scanner (*top*) and colorimetric detector (*bottom*). Mineralization nodules were visualized using a light microscope (**C**). The intensity of mineralized nodule formation was quantified by eluting ARS stains with DMSO, and measured by the Multiskan GO microplate spectrophotometer (**D**). Data represent the mean ± S.E.M. of experiments. *, *p* < 0.05: statistically significant difference when compared to the control. #, *p* < 0.05: statistically significant difference when compared to OS.

To further demonstrate the effects of 7-HYB in osteoblast differentiation, Alizarin red S (ARS) staining was performed to detect the late differentiation of preosteoblasts and we observed the degree of matrix mineralization using a scanner and colorimetric detector at seven and 14 days. The mineralized nodule was formed at 14 days, ARS staining exhibited that 7-HYB promoted the late osteoblasts differentiation in a dose dependent manner (Figure 3A,B). To confirm the observation, we visualized and quantified ARS staining. The results revealed that the mineralized nodule formation was significantly increased by 7-HYB in a dose-dependent manner (Figure 3C,D).

### 2.4. 7-HYB Increases Cell Migration in Osteoblast Differentiation of Preosteoblasts

**Figure 4:**
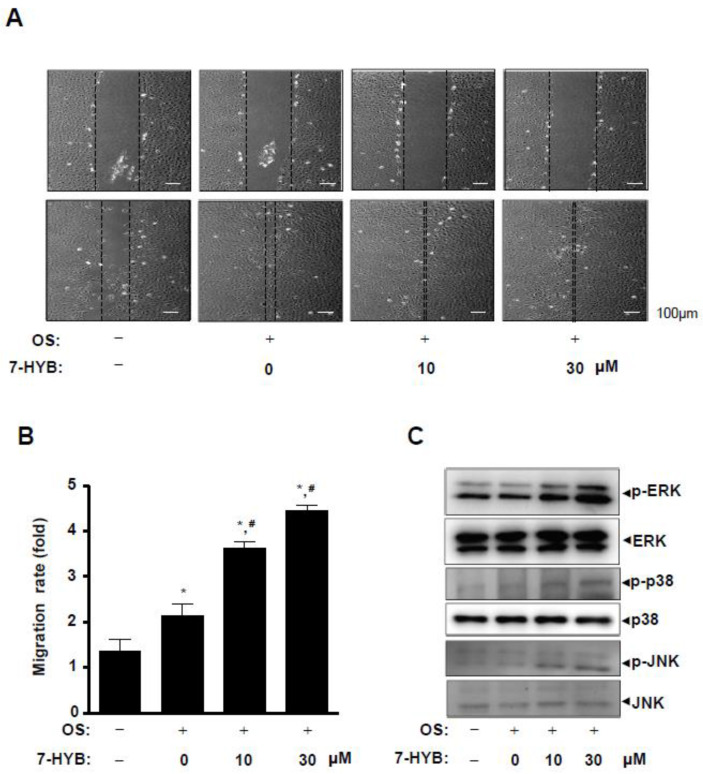
Effect of 7-HYB on cell migration and MAPKs signaling during osteoblast differentiation. (**A**,**B**) After preosteoblasts were cultured in OS with 7-HYB for 24 h, cell migration was observed under a light microscope at 0 h (*top*) and 24 h (*bottom*) (**A**), and cell migration rate (fold) was expressed as a bar graph normalized to the control (**B**). (**C**) Phospho (p)-ERK, ERK2, p-p38, p38, p-JNK, and JNK were assessed by Western blot analysis. Data represent the mean ± S.E.M. of experiments. *, *p* < 0.05: statistically significant difference when compared to the control. #, *p* < 0.05: statistically significant difference when compared to OS.

We next asked whether cell migration could be regulated by 7-HYB during osteoblast differentiation. In wound healing migration assay, the induction of osteoblast differentiation increased cell migration rate, and the closure rate of the cells forward the wound area was significantly accelerated by the treatment of 7-HYB in a dose dependent manner (Figure 4A,B). Under the same condition, we subsequently examined the involvement of mitogen-activated protein kinases (MAPKs) in 7-HYB-mediated cell migration. 7-HYB obviously increased the phosphorylation of ERK1/2, p38, and JNK (Figure 4C).

### 2.5. 7-HYB Stimulates BMP2-Smad1/5/8-RUNX2 and β-catenin Signaling in Osteoblast Differentiation

**Figure 5:**
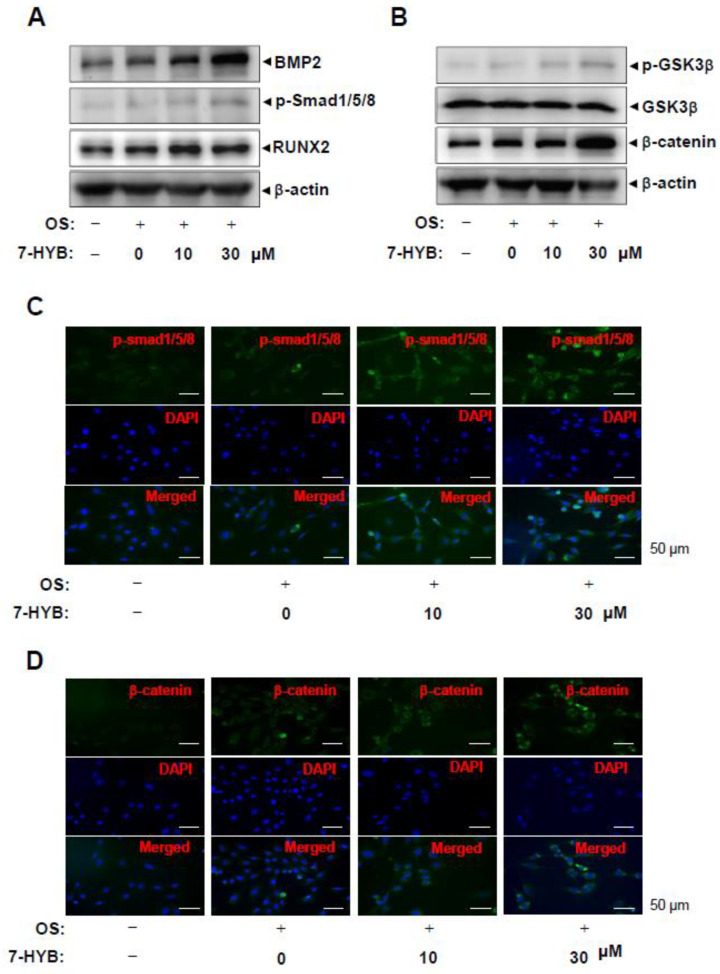
Effect of 7-HYB on the BMP2 and β-catenin pathways during osteoblast differentiation. (**A**,**B**) After preosteoblasts were cultured with OS with 7-HYB for 24 h, BMP2, p-Smad1/5/8, RUNX2, and β-actin (**A**), and p-GSK3β, GSK3β, β-catenin, and β-actin (**B**) were assessed by Western blot analysis. β-actin was used as a loading control. (**C**,**D**) After 24 h, p-Smad1/5/8 was immunostained with rabbit anti-p-Smad1/5/8 antibody and Alexa-Fluor 488-conjugated secondary antibody (*green*). Then, the cells were counterstained with DAPI (*blue*). The *bottom panels* show the merged images of the *top* and *middle panels*.

To further elucidate the mechanisms underlying the stimulatory effects of 7-HYB on the differentiation of preosteoblasts, bone morphogenetic protein (BMP)-Smad1/5/8-RUNX2 signaling was examined during osteoblast differentiation. 7-HYB increased the level of BMP2 protein, the phosphorylation of Smad1/5/8 protein, and the expression of RUNX2, which is a key transcription factor during osteoblast differentiation (Figure 5A,C). We also investigated β-catenin signaling during osteoblast differentiation. These result revealed increases in the phosphorylation of GSK3β and the level in response to the treatment of 7-HYB (Figure 5B,D).

### 2.6. 7-HYB Does Not Affect the Cell Toxicity in Bone Marrow Macrophages (BMMs), Premature Osteoclasts, and Mature Osteoclasts

**Figure 6:**
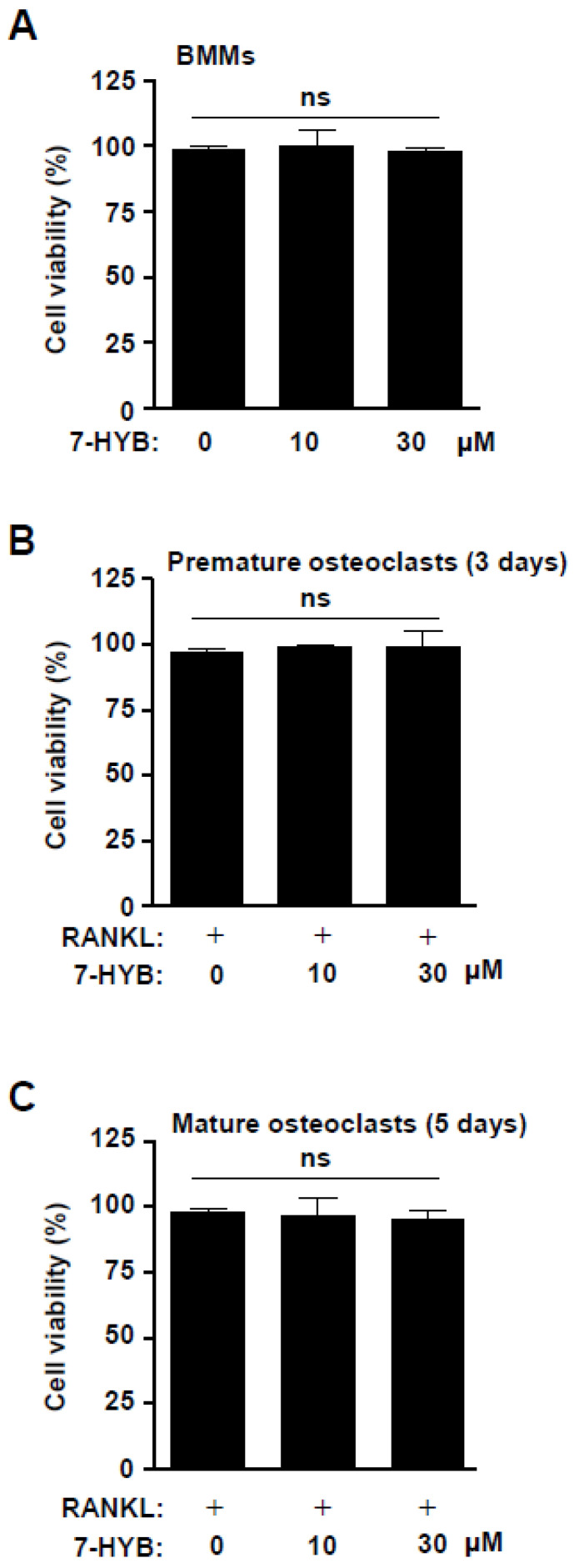
Effect of 7-HYB on the cytotoxicity of osteoclast lineages. (**A**) Bone marrow macrophages (BMMs) were treated with 7-HYB (10 and 30 μM) for 24 h. (**B**,**C**) BMMs were differentiated into osteoclasts with RANKL (100 ng/mL) in the absence and presence of 7-HYB (10 and 30 μM) for three days (**B**) and five days (**C**). Cell viability was measured using the MTT assay. Data represent the mean ± S.E.M. of experiments. ns: no significant difference when compared to the control.

To assess the biological effects of 7-HYB on osteoclastogenesis, we firstly examined cell viability in BMMs, preosteoclasts, and mature osteoclasts. At concentrations ranging from 10 to 30 μM of 7-HYB, no cytotoxic effects were observed in the BMMs (Figure 6A), and under RANKL-induced osteoclast differentiation at three days (premature osteoclasts) and five days (mature osteoclasts) (Figure 6B,C).

### 2.7. 7-HYB Has No Biological Activities on TRAP-Positive Multinucleated Osteoclasts (MNCs) and Gene Expression in RANKL-Induced Osteoclastogenesis

**Figure 7:**
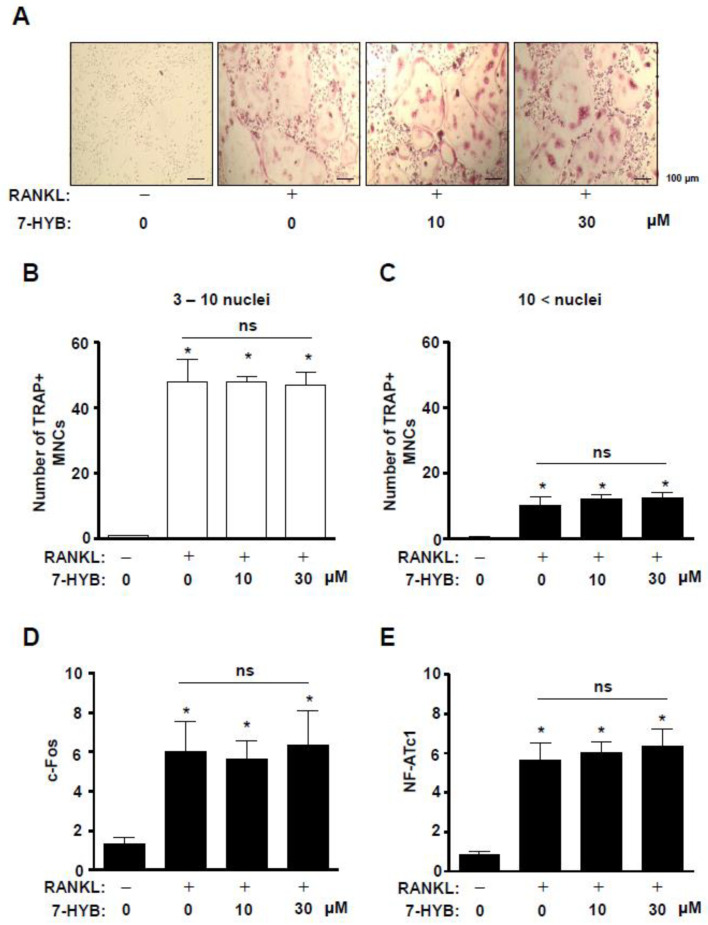
Effect of 7-HYB on TRAP-positive multinucleated osteoclasts (MNCs) and gene expression in RANKL-induced osteoclastogenesis. (**A**–**C**) BMMs were cultured in M-CSF (30 ng/mL) and RANKL (100 ng/mL) with 7-HYB (10 and 30 μM) for five days. TRAP staining were observed using a light microscope (**A**), and the numbers of TRAP-positive MNCs with 3–10 nuclei (**B**) and > 10 nuclei (**C**) were counted. Scale bar: 200 μm. (**D**,**E**) After BMMs were cultured in M-CSF (30 ng/mL) and RANKL (100 ng/mL) with 7-HYB (10 and 30 μM) for three days, c-Fos (**D**) and NF-ATc1 (**E**) were analyzed by qRT-PCR and the values obtained for the target gene expression were expressed as a bar graph. Data represent the mean ± S.E.M. of experiments. *, *p* < 0.05: statistically significant difference when compared to the control. ns: no significant difference when compared to RANKL.

We next investigated the effects of 7-HYB on osteoclastogenesis of BMMs. After BMMs were incubated with RANKL in the absence and presence of 7-HYB at concentrations of 10 and 30 μM for five days, osteoclast differentiation was detected by using TRAP assays. As shown in Figure 7A–C, 7-HYB did not affect TRAP staining, and TRAP-positive multinucleated osteoclasts (MNCs) compared to the number of 3–10 nuclei and 10 < nuclei. We further examined whether 7-HYB influenced osteoclast-related gene expression in RANKL-induced osteoclastogenesis. The results also showed that 7-HYB did not affect gene expression of c-Fos and NF-ATc1 (Figure 7D,E).

## 3. Discussion

Osteoblast lineage cells play a critical role in the bone formation mainly through three steps: Proliferation, when preosteoblasts increase numerically, differentiation, when preosteoblasts become osteoblasts, and matrix mineralization when mature osteoblasts form new bone matrix [2]. Dysregulation in these steps is one of pathogenesis in bone diseases such as osteoporosis [2]. In the present study, we first found the biological effects of 7-HYB in preosteoblast. 7-HYB potentiated the expression and enzymatic activity of ALP, and enhanced mineralized nodule formation during differentiation of preosteoblasts. It was reported that ALP activity is an early differentiation marker of osteoblast lineage cells, which induces and regulates specific osteoblast genes. Mature osteoblasts subsequently form matrix mineralization by calcium deposition [16,17,18]. Therefore, the findings of this study indicate that 7-HYB increases the early and late differentiation of preosteoblasts, leading to differentiation into mature osteoblasts responsible for bone formation.

During the recent years, several growth factors with positive effects on osteoblast linage cells have been identified [8]. Among these factors, BMPs have long been recognized for their function to increase the differentiation of osteoblast linage cells [19,20]. Especially, BMP2 induces bone formation in osteoblasts linage cells via interaction with BMP receptor IA (BMPRIA) or BMPRIB, and BMPRII [21]. In the present study, the treatment of 7-HYB enhanced the level of BMP2 protein. It was also reported that BMP2- signaling activates Smad1/5/8 and forms complexes between Smad1/5/8 and Smad4. The complexes are translocated into nucleus and induce the transcription of RUNX2 that is a key transcription factor in the differentiation of osteoblast linage cells [21,22,23]. Thus, we also investigated BMP2 signaling molecules and our results demonstrated that 7-HYB obviously increases the phosphoryation of Smad1/5/8 and the expression of RUNX2. The signaling pathway also induces ALP expression during differentiation of osteoblasts lineage cells [24,25]. These data suggest that 7-HYB regulates osteoblast differentiation via BMP2 signaling.

Several studies have demonstrated that RUNX2 combines BMP2 signaling with Wnt/β-catenin signaling, and BMP2 expression is also activated by canonical Wnt/β-catenin signaling in osteoblast lineage cells [2,26,27,28,29]. It has indicated that Wnt/β-catenin signaling plays an important role in osteoblast differentiation, particularly in bone mineralization, remodeling, and maintenance [2,30]. It was also reported that Wnt/β-catenin signaling is anabolic for bone formation [8,18,31,32,33]. In the present study, we demonstrated that 7-HYB also increased the phosphorylation of GSK3β and the expression level of β-catenin. As previously reported that canonical Wnt/β-catenin signaling induces phosphorylation of GSK3β leading to its inactivation, and it consequently stabilizes, and accumulates, and translocates β-catenin proteins into nucleus to regulate gene transcription [34,35,36]. Thus, the findings of this study suggest that 7-HYB activates BMP2 and Wnt/β-catenin signaling to enhance osteoblast differentiation and matrix mineralization.

Bone formation, remodeling, and maintenance are a physiologically complex process, and a series of events occur including cell migration of osteoblast lineage cells [37,38]. It is well known that the Wnt/β-catenin signaling pathway participates in the regulation of cell migration and differentiation [28,39,40]. The non-canonical BMP2 signaling pathway also is involved in the regulation of cell migration and differentiation in osteoblast lineage cells through the activation of MAPKs such as ERK1/2, p38, and JNK1/2 [41,42,43,44]. In the present study, we found that 7-HYB increased cell migration during the differentiation of preosteoblasts. In addition, 7-HYB activates MAPKs including ERK1/2, p38. Thus, these results suggest that 7-HYB promotes cell migration and differentiation through the BMP2 and Wnt/β-catenin signaling pathways.

In conclusion, we first demonstrated that 7-HYB isolated from the seeds of *Myristica fragrans* plays an important role in migration, differentiation, and mineralization in osteoblasts. Natural compounds have been used to treat a variety of disease and have increasingly attracted interest in the treatment and prevention of bone diseases [2,45,46]. Recently, there was the rapid evolution of laser technology in dentistry, and laser therapy has shown beneficial effects [47,48,49]. Thus, the combination of natural compounds and laser therapy could be a possible clinical approach. Furthermore, our finding provides that 7-HYB might be useful sources for development of new drugs that could be used to treat bone diseases such as osteoporosis and periodontal disease.

## 4. Materials and Methods

### 4.1. Extraction and Isolation of 7-((7S)-8′-(benzo[3′,4′]dioxol-1′-yl)-7-hydroxypropyl)benzene-2,4-diol

The seeds of *Myristica fragrans* Houtt. were purchased from the commercial herbal market Humanherb, Gyeongsan, Republic of Korea. The seeds of *M. fragrans* Houtt. (2 kg) were extracted with MeOH for 2 h (3 × 2 L). The crude MeOH extracts (96 g) were suspended in distilled water (2 L) and subsequently solvent was partitioned using Hx, EtOAc, and BuOH. The EtOAc soluble fraction (54 g) was subjected to silica gel (Kieselgel 60, 70–230 mesh, Merck, Germany) column chromatography and eluted with a gradient of Hx and EtOAc (20:1 to 1:1) to collect sixteen fractions (MFE 1 ~ MFE 16). The MFE 7 (10.1 g) was subjected to silica gel column chromatography eluted with Hx and EtOAc (5:1 to 0:1) to afford two subfractions (MFE 7-1 ~ MFE 7-2). Subfraction MFE 7-2 (2.5 g) was further purified on a reversed phase (ODS-A) column chromatography eluted with a gradient of MeOH and H_2_O (1:1 to 5:1) to give an active compound (50 mg). The active compound was identified and characterized as ((7S)-8′-(benzo[3′,4′]dioxol-1′-yl)-7-hydroxypropyl)benzene-2,4-diol (7-HYB) by comparison of their various spectroscopic data with before literature [13].

### 4.2. ((7S)-8′-(benzo[3′,4′]dioxol-1′-yl)-7-hydroxypropyl)benzene-2,4-diol (7-HYB)

Colorless oil; ESI-MS *m*/*z* = 287.09 [M-H]^-^, molecular formula C_16_H_16_O_5_; ^1^H-NMR (500 MHz, CDCl_3_) *δ* 6.89 (1H, d, *J* = 1.2 Hz, H-2′), 6.88 (1H, dd, *J* = 1.2, 8.0 Hz, H-6′), 6.87 (1H, d, *J* = 8.4 Hz, H-6), 6.79 (1H, d, *J* = 8.0 Hz, H-5′), 6.33 (1H, dd, *J* = 2.4, 8.4 Hz, H-5), 6.28 (1H, d, *J* = 2.4 Hz, H-3), 5.93 (2H, d, *J* = 2.4 Hz, OCH_2_O), 4.89 (1H, dd, *J* = 2.4, 10.4 Hz, H-7), 2.84 and 2.64 (each 1H, m, H-8′), 2.05 and 1.90 (each 1H, m, H-8); ^13^C-NMR (125 MHz, CDCl_3_) *δ* 157.7 (C-4), 157.1 (C-2), 149.2 (C-3′), 148.6 (C-4′), 137.5 (C-1), 131.0 (C-6), 120.6 (C-6′), 114.3 (C-1′), 109.2 (C-5), 109.0 (C-5′), 107.6 (C-2′), 104.2 (C-3), 102.4 (OCH_2_O), 79.1 (C-7), 31.6 (C-8), 25.4 (C-8′).

### 4.3. Nuclear Magnetic Resonance (NMR)

Nuclear magnetic resonance (NMR) experiments were performed on a JEOL ECX-500 spectrometer, operating at 500 MHz for ^1^H and 125 MHz for ^13^C NMR spectrum (JEOL Ltd., Akishima, Japan). All chemical shifts were referenced relative to the corresponding signals (δ_H_ 3.31/δ_C_ 49.15 for CD_3_OD). Electron ionization mass spectrometer (EI-MS) data were obtained using micromass spectrum (AUTOSPEC, Glasgow, UK). High performance liquid chromatography (HPLC) was performed using Agilent 1200 series (Agilent Technologies, CA, USA). The silica gel 60 (Merck 230–400 mesh, ASTM, Germany) and ODS-A (Merck ASTM, Germany) were used for column chromatography.

### 4.4. Culture of MC3T3E-1 Preosteoblasts, and Differentiation of Osteoblasts

MC3T3E-1 preosteoblasts (#CRL-2593) purchased from the American Type Culture Collection (ATCC) (Manassas, VA, USA) were kindly provided by Bioevaluation Center (Korea Research Institute of Bioscience and Biotechnology, Republic of Korea). The cells were cultured in *α*-minimum essential medium (*α*-MEM) without L-ascorbic acid (WELGEME, Inc., Seoul, Republic of Korea) supplemented with 10% fetal bovine serum (FBS), penicillin (100 units/mL), and streptomycin (100 μg/mL) at 37 °C in a humidified atmosphere of 5% CO_2_ and 95% air. Osteoblast differentiation was induced by changing to osteogenic supplement medium (OS) containing 50 μg/mL L-ascorbic acid (L-AA) and 10 mM β-glycerophosphate (β-GP) (Sigma-Aldrich, St. Louis, MO, USA). The medium was replaced every two days during the incubation period as previously described [50].

### 4.5. MTT Assay

Cell viability was measured using an 3-[4,5-dimethylthiazol-2-yl]-2,5-diphenyltetrazolium bromide (MTT) assay to detect NADH-dependent dehydrogenase activity as previously described [51].

### 4.6. Western Blot Analysis

Western blot analysis was carried out as previously described [52]. Briefly, equal amounts of proteins (20 μg) transferred to a polyvinylidene fluoride (PVDF) membrane (Millipore, Bedford, MA) were blocked for 1 h at room temperature and incubated overnight at 4 °C with the primary antibodies. The membrane incubated with diluted horseradish peroxidase (HRP)-conjugated secondary antibodies (1:10,000, Jackson ImmunoResearch, West Grove, PA, USA) for 2 h at room temperature was detected using the ProteinSimple detection system (ProteinSimple Inc., Santa Clara, CA, USA).

### 4.7. Cell Migration Assay

Cell migration was accessed using an in vitro wound healing assay as previously described [53]. Briefly, the cells were wounded with a 200 μL pipette tip and cultured in the absence and presence of 7-HYB for 24 h at 37 °C in a humidified atmosphere of 5% CO_2_ and 95% air. Cell migration was observed under light microscopy and cell migration rate was quantified.

### 4.8. Alkaline Phosphatase (ALP) Staining Assay

Cells were washed with 1 × PBS and then fixed in 10% formalin for 15 min at room temperature. After washing with distilled water, the cells were incubated with substrate solution for the reaction of ALP at 37 °C for 1 h, followed according to the manufacturer’s protocol (Takara Bio Inc., Shiga, Japan) as previously described [50].

### 4.9. ALP Activity Assay

The cell lysates were performed according to the manufacturer’s protocol using alkaline phosphatase activity colorimetric assay kit (Biovision, Milpitas, CA, USA) as previously described [50]. The absorbance was measured at 405 nm using the Multiskan GO microplate spectrophotometer (Thermo Fisher Scientific, Waltham, MA, USA).

### 4.10. Alizarin Red S (ARS) Staining

Cells were fixed in 10% formalin for 15 min and rinsed with distilled water. Cells were stained with 2% ARS (pH 4.2) (Sigma-Aldrich) for 10 min with gentle agitation. The level of ARS staining was observed using a scanner and colorimetric detector (ProteinSimple Inc., Santa Clara, CA, USA). After scanning the stained wells, stains were dissolved in 100% DMSO and the absorbance was measured at 590 nm using the Multiskan GO Microplate Spectrophotometer (Thermo Fisher Scientific).

### 4.11. Immunocytochemistry

Immunocytochemistry was performed as previously described [53]. Briefly, the cells were blocked with 3% BSA diluted in PBS for 1 h and incubated with specific primary antibodies overnight at 4 °C. Subsequently, the cells were incubated with an antirabbit secondary antibody labeled with Alexa-Fluor 488 (1:500 dilution, Invitrogen, Carlsbad, CA, USA) for 2 h at room temperature. Next, the cells were incubated with 4′,6-diamidino-2-phenylindole (DAPI) (Sigma-Aldrich) for 10 min at room temperature. The cells were washed three times, mounted, and viewed on a confocal microscope (K1-Fluo Confocal Laser Scanning Microscope, Republic of Korea).

### 4.12. Live Subject Statement

All mice used in this study were maintained in accordance with the National Institute of Toxicological Research of the Korea Food and Drug Administration guidelines for the humane care and use of laboratory animals. All protocols in the current study were approved by the Chungbuk National University Institutional Animal Care and Use Committee (IACUC) (CBNUA-792-15-01) and complied with the Korean National Institute of Health Guide for the Care and Use of Laboratory Animals.

### 4.13. Culture of Bone Marrow Macrophages, and Osteoclast Differentiation

Mouse bone marrow cells isolated from five-week-old mice were cultured dishes in α-MEM (WELGEME) containing 10% FBS), penicillin (100 units/mL), and streptomycin (100 μg/mL) at 37 °C overnight in a humidified atmosphere of 5% CO_2_ and 95% air. The next day, adherent cells were discarded, and floating cells were further incubated with M-CSF (30 ng/mL) on Petri dishes. After three days, BMMs became adherent, and then the cells were incubated with RANKL (100 ng/mL) and M-CSF (30 ng/mL) until five days to induce osteoclast differentiation.

### 4.14. Tartrate-Resistant Acid Phosphatase (TRAP) Staining

BMMs were differentiated into osteoclasts for five days, fixed with 10% formalin for 30 min, and washed with 1 X PBS. Then, the cells were stained for TRAP according to the manufacturer’s protocol (Takara Bio Inc.). The TRAP-positive multinucleated cells (MNCs) were counted as mature osteoclasts using a light microscope.

### 4.15. Quantitative Real-Time Polymerase Chain Reaction (PCR) Analysis

Total RNA was extracted using the RNAqueous^®^ kit and cDNA synthesized from RNA (1 µg) using the high-capacity RNA-to-cDNA kit (Applied Biosystems, Foster City, CA, USA) according to the manufacturer’s protocol. Quantitative real-time PCR was performed using a 7500 Real-Time PCR System (Applied Biosystems).

### 4.16. Statistical Analysis

The data were analyzed using Prism Version 5 program (GraphPad Software, Inc., San Diego, CA). All numeric values are presented as the means ± S.E.M. The statistical significance of data was determined using a Student’s unpaired *t* test. A value of *p* < 0.05 was considered to indicate statistical significance.

## References

[1] Karsenty G. (2003). The complexities of skeletal biology. Nature.

[2] An J., Yang H., Zhang Q., Liu C., Zhao J., Zhang L., Chen B. (2016). Natural products for treatment of osteoporosis: The effects and mechanisms on promoting osteoblast-mediated bone formation. Life Sci..

[3] Seeman E. (2002). Pathogenesis of bone fragility in women and men. Lancet.

[4] Khosla S., Riggs B.L. (2005). Pathophysiology of age-related bone loss and osteoporosis. Endocrinol. Metab. Clin. N. Am..

[5] Marie P.J. (2015). Osteoblast dysfunctions in bone diseases: From cellular and molecular mechanisms to therapeutic strategies. Cell. Mol. Life Sci..

[6] Vondracek S.F., Minne P., McDermott M.T. (2008). Clinical challenges in the management of osteoporosis. Clin. Interv. Aging.

[7] Kawai M., Modder U.I., Khosla S., Rosen C.J. (2011). Emerging therapeutic opportunities for skeletal restoration. Nat. Rev. Drug Discov..

[8] Marie P.J., Kassem M. (2011). Osteoblasts in osteoporosis: Past, emerging, and future anabolic targets. Eur. J. Endocrinol..

[9] Adjene J.O., Nwose E.U. (2010). Histological effects of long term consumption of nutmeg on the medial geniculate body of adult Wistar rats. N. Am. J. Med. Sci..

[10] Barceloux D.G. (2009). Nutmeg (Myristica fragrans Houtt.). Dis. Mon..

[11] Van Gils C., Cox P.A. (1994). Ethnobotany of nutmeg in the Spice Islands. J. Ethnopharmacol..

[12] Abourashed E.A., El-Alfy A.T. (2016). Chemical diversity and pharmacological significance of the secondary metabolites of nutmeg (Myristica fragrans Houtt.). Phytochem. Rev..

[13] Cuong T.D., Hung T.M., Na M., do T.H., Kim J.C., Lee D., Ryoo S., Lee J.H., Choi J.S., Min B.S. (2011). Inhibitory effect on NO production of phenolic compounds from Myristica fragrans. Bioorg. Med. Chem. Lett..

[14] Duan L., Tao H.W., Hao X.J., Gu Q.Q., Zhu W.M. (2009). Cytotoxic and antioxidative phenolic compounds from the traditional Chinese medicinal plant, Myristica fragrans. Planta Med..

[15] El-Alfy A.T., Abourashed E.A., Patel C., Mazhari N., An H., Jeon A. (2019). Phenolic compounds from nutmeg (Myristica fragrans Houtt.) inhibit the endocannabinoid-modulating enzyme fatty acid amide hydrolase. J. Pharm. Pharmacol..

[16] Guntur A.R., Rosen C.J. (2011). The skeleton: A multi-functional complex organ: New insights into osteoblasts and their role in bone formation: The central role of PI3Kinase. J. Endocrinol..

[17] Lee H.S., Jung E.Y., Bae S.H., Kwon K.H., Kim J.M., Suh H.J. (2011). Stimulation of osteoblastic differentiation and mineralization in MC3T3-E1 cells by yeast hydrolysate. Phytother. Res..

[18] Kim M.B., Song Y., Hwang J.K. (2014). Kirenol stimulates osteoblast differentiation through activation of the BMP and Wnt/beta-catenin signaling pathways in MC3T3-E1 cells. Fitoterapia.

[19] Katagiri T., Yamaguchi A., Komaki M., Abe E., Takahashi N., Ikeda T., Rosen V., Wozney J.M., Fujisawa-Sehara A., Suda T. (1994). Bone morphogenetic protein-2 converts the differentiation pathway of C2C12 myoblasts into the osteoblast lineage. J. Cell Biol..

[20] Wozney J.M., Rosen V., Celeste A.J., Mitsock L.M., Whitters M.J., Kriz R.W., Hewick R.M., Wang E.A. (1988). Novel regulators of bone formation: Molecular clones and activities. Science.

[21] Miyazono K., Kamiya Y., Morikawa M. (2010). Bone morphogenetic protein receptors and signal transduction. J. Biochem..

[22] Canalis E., Economides A.N., Gazzerro E. (2003). Bone morphogenetic proteins, their antagonists, and the skeleton. Endocr. Rev..

[23] Lee M.H., Kim Y.J., Kim H.J., Park H.D., Kang A.R., Kyung H.M., Sung J.H., Wozney J.M., Ryoo H.M. (2003). BMP-2-induced Runx2 expression is mediated by Dlx5, and TGF-beta 1 opposes the BMP-2-induced osteoblast differentiation by suppression of Dlx5 expression. J. Biol. Chem..

[24] Gaur T., Lengner C.J., Hovhannisyan H., Bhat R.A., Bodine P.V., Komm B.S., Javed A., van Wijnen A.J., Stein J.L., Stein G.S. (2005). Canonical WNT signaling promotes osteogenesis by directly stimulating Runx2 gene expression. J. Biol. Chem..

[25] Phimphilai M., Zhao Z., Boules H., Roca H., Franceschi R.T. (2006). BMP signaling is required for RUNX2-dependent induction of the osteoblast phenotype. J. Bone Miner. Res..

[26] Rawadi G., Vayssiere B., Dunn F., Baron R., Roman-Roman S. (2003). BMP-2 controls alkaline phosphatase expression and osteoblast mineralization by a Wnt autocrine loop. J. Bone Miner. Res..

[27] Fukuda T., Kokabu S., Ohte S., Sasanuma H., Kanomata K., Yoneyama K., Kato H., Akita M., Oda H., Katagiri T. (2010). Canonical Wnts and BMPs cooperatively induce osteoblastic differentiation through a GSK3beta-dependent and beta-catenin-independent mechanism. Differentiation.

[28] Zhang R., Oyajobi B.O., Harris S.E., Chen D., Tsao C., Deng H.W., Zhao M. (2013). Wnt/beta-catenin signaling activates bone morphogenetic protein 2 expression in osteoblasts. Bone.

[29] Zhang J.F., Li G., Chan C.Y., Meng C.L., Lin M.C., Chen Y.C., He M.L., Leung P.C., Kung H.F. (2010). Flavonoids of Herba Epimedii regulate osteogenesis of human mesenchymal stem cells through BMP and Wnt/beta-catenin signaling pathway. Mol. Cell Endocrinol..

[30] Day T.F., Guo X., Garrett-Beal L., Yang Y. (2005). Wnt/beta-catenin signaling in mesenchymal progenitors controls osteoblast and chondrocyte differentiation during vertebrate skeletogenesis. Dev. Cell.

[31] Lo Y.C., Chang Y.H., Wei B.L., Huang Y.L., Chiou W.F. (2010). Betulinic acid stimulates the differentiation and mineralization of osteoblastic MC3T3-E1 cells: Involvement of BMP/Runx2 and beta-catenin signals. J. Agric. Food Chem..

[32] Bodine P.V., Komm B.S. (2006). Wnt signaling and osteoblastogenesis. Rev. Endocr. Metab. Disord..

[33] Qiu W., Andersen T.E., Bollerslev J., Mandrup S., Abdallah B.M., Kassem M. (2007). Patients with high bone mass phenotype exhibit enhanced osteoblast differentiation and inhibition of adipogenesis of human mesenchymal stem cells. J. Bone Miner. Res..

[34] MacDonald B.T., He X. (2012). Frizzled and LRP5/6 receptors for Wnt/beta-catenin signaling. Cold Spring Harb. Perspect. Biol..

[35] Reya T., Clevers H. (2005). Wnt signalling in stem cells and cancer. Nature.

[36] Moon R.T., Bowerman B., Boutros M., Perrimon N. (2002). The promise and perils of Wnt signaling through beta-catenin. Science.

[37] Ghiasi M.S., Chen J., Vaziri A., Rodriguez E.K., Nazarian A. (2017). Bone fracture healing in mechanobiological modeling: A review of principles and methods. Bone Rep..

[38] Enoki Y., Sato T., Kokabu S., Hayashi N., Iwata T., Yamato M., Usui M., Matsumoto M., Tomoda T., Ariyoshi W. (2017). Netrin-4 Promotes Differentiation and Migration of Osteoblasts. In Vivo.

[39] Wang Y., Chen H., Zhang H. (2019). Kaempferol promotes proliferation, migration and differentiation of MC3T3-E1 cells via up-regulation of microRNA-101. Artif. Cells Nanomed. Biotechnol..

[40] Kim W., Kim M., Jho E.H. (2013). Wnt/beta-catenin signalling: From plasma membrane to nucleus. Biochem. J..

[41] Westhoff M.A., Serrels B., Fincham V.J., Frame M.C., Carragher N.O. (2004). SRC-mediated phosphorylation of focal adhesion kinase couples actin and adhesion dynamics to survival signaling. Mol. Cell. Biol..

[42] Chang Y.M., Shih Y.T., Chen Y.S., Liu C.L., Fang W.K., Tsai C.H., Tsai F.J., Kuo W.W., Lai T.Y., Huang C.Y. (2011). Schwann Cell Migration Induced by Earthworm Extract via Activation of PAs and MMP2/9 Mediated through ERK1/2 and p38. Evid. Based Complement. Altern. Med..

[43] Chen Y.Y., Liu F.C., Chou P.Y., Chien Y.C., Chang W.S., Huang G.J., Wu C.H., Sheu M.J. (2012). Ethanol extracts of fruiting bodies of Antrodia cinnamomea suppress CL1-5 human lung adenocarcinoma cells migration by inhibiting matrix metalloproteinase-2/9 through ERK, JNK, p38, and PI3K/Akt signaling pathways. Evid. Based Complement. Altern. Med..

[44] Liao X., Lu S., Zhuo Y., Winter C., Xu W., Wang Y. (2012). Visualization of Src and FAK activity during the differentiation process from HMSCs to osteoblasts. PLoS ONE.

[45] Soelaiman I.N., Das S., Shuid A.N., Mo H., Mohamed N. (2013). Use of medicinal plants and natural products for treatment of osteoporosis and its complications. Evid. Based Complement. Altern. Med..

[46] Whelan A.M., Jurgens T.M., Bowles S.K. (2006). Natural health products in the prevention and treatment of osteoporosis: Systematic review of randomized controlled trials. Ann. Pharmacother..

[47] Bordea R., Lucaciu O., Câmpian R.S. (2016). Student’s knowledge and opinion regarding the need of implementation of Lasers in Dental Faculty curriculum. Hum. Vet. Med..

[48] Bordea I.R., Lucaciu P.O., Crişan B., Pelekanos S., Câmpian R.S. (2016). The influence f chromophore presence in an experimental bleaching gel on laser assisted tooth whitening efficiency. Studia Ubb Chemia..

[49] Inchingolo F., Tatullo M., Pacifici A., Abenavoli F.M., Pacifici L. (2012). Use of dermal-fat grafts in the post-oncological reconstructive surgery of atrophies in the zygomatic region: Clinical evaluations in the patients undergone to previous radiation therapy. Head Face Med..

[50] Park K.R., Lee H., Cho M., Yun H.M. (2020). A Phytochemical Constituent, (E)-Methyl-Cinnamate Isolated from Alpinia katsumadai Hayata Suppresses Cell Survival, Migration, and Differentiation in Preosteoblasts. Int. J. Mol. Sci..

[51] Park K.R., Yun H.M. (2019). RANKL-induced osteoclastogenesis in bone marrow-derived macrophages is suppressed by cisapride. Toxicology.

[52] Park K.R., Kim E.C., Hong J.T., Yun H.M. (2018). Dysregulation of 5-hydroxytryptamine 6 receptor accelerates maturation of bone-resorbing osteoclasts and induces bone loss. Theranostics.

[53] Park K.R., Yun H.M., Hong J.T. (2020). G721-0282 inhibits cell growth and induces apoptosis in human osteosarcoma through down-regulation of the STAT3 pathway. Int. J. Biol. Sci..

